# The Relationship between Simple Snoring and Sleep Bruxism: A Polysomnographic Study

**DOI:** 10.3390/ijerph17238960

**Published:** 2020-12-02

**Authors:** Monika Michalek-Zrabkowska, Mieszko Wieckiewicz, Piotr Macek, Pawel Gac, Joanna Smardz, Anna Wojakowska, Rafal Poreba, Grzegorz Mazur, Helena Martynowicz

**Affiliations:** 1 Department of Internal Medicine, Occupational Diseases, Hypertension and Clinical Oncology, Wroclaw Medical University, 213 Borowska St., 50-556 Wroclaw, Poland; monika.michalek1@gmail.com (M.M.-Z.); macekpiotr@op.pl (P.M.); ania.wojakowska@wp.pl (A.W.); sogood@poczta.onet.pl (R.P.); grzegorzmaz@yahoo.com (G.M.); helenamar@poczta.onet.pl (H.M.); 2 Department of Experimental Dentistry, Wroclaw Medical University, 26 Krakowska St., 50-425 Wroclaw, Poland; joannasmardz1@gmail.com; 3 Department of Hygiene, Wroclaw Medical University, 7 Mikulicza-Radeckiego St., 50-345 Wroclaw, Poland; pawelgac@interia.pl

**Keywords:** simple snoring, sleep bruxism, polysomnography

## Abstract

Simple snoring is defined as the production of sound in the upper aerodigestive tract during sleep, not accompanied by other pathologies. Sleep bruxism (SB) refers to repetitive phasic, tonic, or mixed masticatory muscle activity during sleep. In this study, we investigated the relationship between simple snoring and SB in patients without obstructive sleep apnea (OSA). A total of 565 snoring subjects underwent polysomnography. After examination, individuals with OSA were excluded from the study group. Finally, 129 individuals were analyzed. The bruxism episode index was positively correlated with maximum snore intensity. Phasic bruxism was positively correlated with snore intensity in all sleep positions. Bruxers had a significantly decreased average and minimum heart rate compared with non-bruxers. Supine sleep position seemed to have a significant impact on snore intensity and SB. In summary, our study showed the relationship between SB, snore intensity, and body position. Phasic bruxism was positively correlated with snore intensity despite the body position, which is an interesting and novel finding.

## 1. Introduction

Snoring is defined as the production of sound due to the vibration of respiratory structures in the upper aerodigestive tract during sleep. Simple snoring (SS) not accompanied by daytime sleepiness and fatigue or obstructive sleep apnea (OSA) is called primary snoring [1]. Simple snoring is also known as non-apneic snoring [2]. The risk factors for snoring include nasal congestion, obstruction/inflammation of the upper airways, increased body mass index (BMI), male gender, or intake of alcohol, drug, or tobacco [3]. The existing literature pertaining to physical implications of snoring associates this condition with mild symptoms such as dry mouth or irritated tissues and severe symptoms such as excessive daytime sleepiness [4], carotid artery atherosclerosis [5], stroke [6], cardiovascular diseases [7], metabolic syndrome [8], and increased all-cause mortality [9]. On the other hand, a previous study suggested that SS has no adverse effects on an individual [10]. According to the American Academy of Sleep Medicine (AASM), the International Classification of Sleep Disorders, 3rd Edition (ICSD-3) classifies SS as a sleep-related breathing disorder (SRBD) [11]. Some authors have suggested and developed methods for the assessment of snoring. For instance, Lee et al. defined a snore sound as “an obvious deflection from background (with no minimum decibel threshold), which was in phase with inspiration and occurred during sleep” [5]. They considered three or more snore events in a 30 s epoch as a “snore epoch,” and subsequently calculated the snore index. The criteria based on snore intensity with peak ≥40 dB for ≥30% sleep breaths were established by Guzman et al. [12]. The definition of snore trains is used to describe periods with multiple single-snore events [13]. The epidemiology of SS is not clearly explained in the literature but depending on the method of measurement and the population studied, its incidence varies between 2% and 85% [3]. Among the studies that focus on snoring, the Hungarian population survey by Torzsa et al. is worth mentioning as it is the largest study to analyze the prevalence of snoring, in which 37% of males and 21% of females reported loud snoring with breathing pauses [14].

Bruxism is a common phenomenon which is defined by the AASM as “repetitive masticatory muscle activity characterized by clenching or grinding of the teeth and/or by bracing or thrusting of the mandible” [11]. This condition may be divided into two types: sleep bruxism (SB) and awake bruxism. According to the international consensus originated by Lobbezoo et al., sleep bruxism is defined as a masticatory muscle activity during sleep that is characterized as either rhythmic (phasic) or nonrhythmic (tonic) and should not be considered as a movement disorder or a sleep disorder in otherwise healthy individuals [15]. The estimated prevalence of sleep bruxism varies with age, ranging from 13% in young adults to 3% in the elderly [15,16]. The consensus proposed distinguishing the risk factors for SB into three groups: biological, psychological, and external. The multifactorial etiology of SB includes genetic vulnerability [17], age, synaptic transmission, disturbances in sleep architecture, perception of stress and anxiety, intake of alcohol and drug, smoking, and comorbidities [16]. Previous studies have acknowledged the following medical conditions as coexisting with SB: OSA [18,19,20], diabetes [19], gastroesophageal reflux disorder [21,22], increased BMI, hypertension [23,24], excessive daytime sleepiness, and snoring [20].

The newest literature-based analysis of the global obstructive sleep apnea prevalence carried out by Benjafield et al. revealed that about “1 billion adults aged 30–69 years were estimated to have OSA, with and without symptoms” [25]. In some countries, the prevalence of OSA even exceeds 50%. Although the relationship between sleep bruxism and obstructive sleep apnea seems to be significant and has also been explored in prior studies, the data obtained are inconsistent. Several studies have confirmed the association between these two conditions [19,26,27,28], whereas some have failed to prove the link between them [29,30,31]. However, in our previous study, we demonstrated the association between OSA and SB [19] and showed a positive correlation among the sleep bruxers having mild or moderate OSA.

Sleep-related breathing disorders are classified based on the differences in the obstruction of the upper airways and in the degrees of alteration in gas exchange during the night. Simple snoring, classified as a separate entity in ICSD-3, is frequently reported by many individuals [32], but its definition and role is not clearly presented in literature. Simple snoring may indicate a slight obstruction in the upper respiratory tract which is a probable risk factor for the development of OSA. OSA is associated with significant morbidity (e.g., diabetes [33], hypertension [34], stroke [35], ischemic heart disease [36], and arrythmias [37,38,39]) as well as mortality [40].

An important question associated with simple snoring and sleep bruxism that remains to be answered is whether these phenomena can coexist and relate. Second, does sleep position impact SB or SS? Therefore, this study aimed to analyze the relationships between SS, SB, and sleep position in patients without OSA.

## 2. Materials and Methods

The study was performed in the Sleep Laboratory of the Department of Internal Medicine, Occupational Diseases, Hypertension and Clinical Oncology at the Wroclaw Medical University (Wroclaw, Poland). The number of *n* = 565 patients included in the study were admitted to the department for polysomnographic examination from the outpatient clinic operating at the Department of Experimental Dentistry of the Wroclaw Medical University, as they reported snoring and were diagnosed with probable bruxism. All the participants provided informed consent to participate in the study. The study was approved by the Ethics Committee of the Wroclaw Medical University (ID KB-794/2019) and was conducted in accordance with the Declaration of Helsinki. Information regarding clinical trial registration is available at www.ClinicalTrials.gov (identifier NCT04214561). After conducting a single, full-night polysomnographic examination, individuals with OSA were excluded from the study. The final study group consisted of 129 individuals, including 38 men and 91 women, who had a mean age of 33.60 ± 9.89 years.

We included the individuals who declared their willingness to participate in the study and fulfilled the following criteria: Aged above 18 years and clinically suspected with SS and/or SB. All participants met ICSD III B 1 or 2 criteria for SB.

Patients who were less than 18 years of age and diagnosed with OSA were excluded from the study. Other exclusion criteria were as follows: inability to undergo polysomnography; secondary bruxism associated with neurological conditions; intake of medicines that affect the neuromuscular functioning; presence of severe mental disorders, active malignancy, neurological disorders, and/or neuropathic pain; coexistence of respiratory insufficiency or active inflammation; addiction to analgesic drugs and/or drugs that affect muscle and breathing functions; and presence of severe allergic symptoms.

SB was assessed according to the AASM standards. Bruxism episodes were qualified as phasic, tonic, or mixed based on the electromyographic (EMG) recording obtained from the masseter muscle region bilaterally, which was complemented by audio and video recording. Bruxism episode index (BEI) indicates the total number of bruxism episodes per hour. To confirm the bruxism episodes, an audiovisual assessment was performed and the EMG measures were taken. According to the AASM standards, the peak of the EMG amplitude during bruxism episode has to be at least twice the amplitude of the background EMG and should not be separated more than 3 s in order to be considered as a part of the same episode [41]. EMG activities were scored as phasic if the episode lasted 2 s with three or more bursts, or as tonic if the episode lasted more than 2 s with sustained bursts, or as mixed if the episode had a mix of them. SB can be confirmed if the value of BEI is at least 2. Based on BEI, we can assess the severity of SB as follows: BEI = 2–4, mild/moderate SB; BEI > 4, severe SB [11].

A full-night polysomnographic assessment was performed using Nox-A1 (Nox Medical, Reykjavik, Iceland) among all patients in the Sleep Laboratory of the Department of Internal Medicine, Occupational Diseases, Hypertension and Clinical Oncology at the Wroclaw Medical University (Wroclaw, Poland). Patient EEGs were recorded using the AASM recommended EEG montages during the PSG. The eight electroencephalographic (EEG) and electrooculographic (EOG) channels were used (F4-M1, C4-M1, O2-M1, F3-M2, C3-M2, O1-M2, E1-M2, E2-M2). The EEG electrode position was determined by an International 10-20 System. Three electrodes have been placed to record chin EMG in mental and submental positioning. Two channels of respiratory effort from respiratory inductance plethysmography (RIP) belts circulating the thorax and abdomen were used. A single modified electrocardiogram Lead II was used to assess ECG. Nasal pressure transducer was used to assess hypopnea. Oronasal thermal airflow sensor was used to assess apnea. Polysomnograms (PSGs) were scored in 30-s epochs. After an automatic analysis was conducted, a manual analysis was performed by certified polysomnographist. Epochs were classified based on the standard criteria for sleep by the AASM 2013 Task Force [42]. The PSG outcomes included the following: sleep latency (SL); rapid eye movement (REM) latency; total sleep time; sleep efficiency; and the ratio of N1 (sleep stage 1), N2 (sleep stage 2), N3 (sleep stage 3), and REM (REM sleep stage). Sleep position was determined automatically using position sensors.

Snoring was monitored using an acoustic sensor and a nasal pressure transducer. All the snore sounds that were in synchrony with breathing and protuberant from the background with an audible oscillatory component were considered as snore events. The Noxturnal software (Nox Medical, Reykjavik, Iceland) recorded a full audio signal with 8.000 Hz sampling. Then, the audio signal was derived and stored with 100 Hz, followed by a conversion with dBc weighting to receive an audio envelop signal in dB. A period of multiple single snores was scored as a snore train. All the snore trains were automatically recorded [13]. Both inspiratory and expiratory snores were scored. Other non-snore sounds—i.e., coughing, groaning, bruxing, duvet, and movement noise, as well as large breathing sounds without any vibration—were excluded.

The statistical package “Dell Statistica 13.1” (Dell Inc., Round Rock, TX, USA) was used to perform statistical analysis. For the quantitative variables, arithmetic means and SDs of the estimated parameters were calculated. The distribution of variables was examined using Lilliefors and W-Shapiro–Wilk tests. For the independent quantitative variables with normal and other than normal distribution, we used Student’s *t*-test and Mann–Whitney *U*-test, respectively. For the dependent quantitative variables with normal distribution, the *t*-test for linked variables was used. In the case of dependent quantitative variables showing the distribution distinct from normal, Wilcoxon’s paired sequence test was applied. The results for qualitative variables were expressed as percentages. For dependent qualitative variables, McNemar’s test or Cochran’s test was used for statistical analysis. To determine the relationship between the analyzed variables, a correlation analysis was performed. The results at the level of *p* < 0.05 were considered statistically significant.

## 3. Results

A total of *n* = 565 patients were included in our study, then after one-night polysomnography patients with OSA (AHI ≥ 5) were excluded. Finally, study group estimated *n* = 129 individuals (mean age 33.60 ± 9.89 years; 38 men and 91 women). The study group had only sleep bruxers and/or snorers. Based on the BEI value calculated from the PSG findings, 98 individuals (75.97%) were diagnosed with SB; of them, 39 (30.23%) met the criteria for mild/moderate bruxism and 59 (69.77%) for severe bruxism.

The polysomnographic parameters calculated for the studied group are presented in Table 1. Among the participants, nine were diagnosed with hypertension, while no one met the criteria for coronary artery disease.

Regarding the mean bruxism and snore parameters differentiated based on body position, BEI supine was higher than BEI non-supine. Phasic bruxism was found to be most frequent bruxism type independent of the sleep position. With respect to snore parameters, snore indexes were found to be higher in the supine position in all sleep stages (Table 2).

In the study group, we found statistically significant differences between severe bruxers (BEI > 4) and the remaining individuals. The maximum snore intensity was found to be significantly higher among the severe bruxers (BEI > 4) than in the group with BEI < 4 (70.83 ± 27.02 dB vs. 60.77 ± 33.88 dB, *p* = 0.05). The mean and maximum snore intensity in the supine position were also significantly higher in individuals with BEI > 4 compared with those with BEI < 4 (mean score intensity: 51.69 ± 27.86 dB vs. 41.57 ± 32.24 dB, *p* = 0.04; maximum snore intensity: 61.70 ± 33.59 dB vs. 49.35 ± 38.65 dB, *p* = 0.05). Furthermore, arousal frequency was observed to be statistically increased in severe bruxers compared with individuals with BEI < 4 (3.82 ± 2.82 *n*/h vs. 2.82 ± 1.87 *n*/h, *p* = 0.02).

To determine the relationship between bruxism, sleep position, polysomnographic parameters, and heart rate, a correlation analysis was subsequently performed (Table 3).

Regardless of bruxism severity, the mean and minimum heart rates were significantly decreased in patients with bruxism (Table 4).

Snore intensity was positively correlated with phasic bruxism in all sleep positions (Table 5).

Based on the impact of body position on bruxism (Table 6), a statistically significant difference was found in BEI total and tonic rates between the supine and non-supine position. The incidence of mixed, phasic, and tonic bruxism episodes was observed more frequently in the supine than in the non-supine position, but a statistically significant difference was found only in the case of tonic bruxism.

Assessing the impact of body position on snoring, snore indices were found to be significantly higher in the supine position compared with the non-supine position (Table 7).

**Figure 1:**
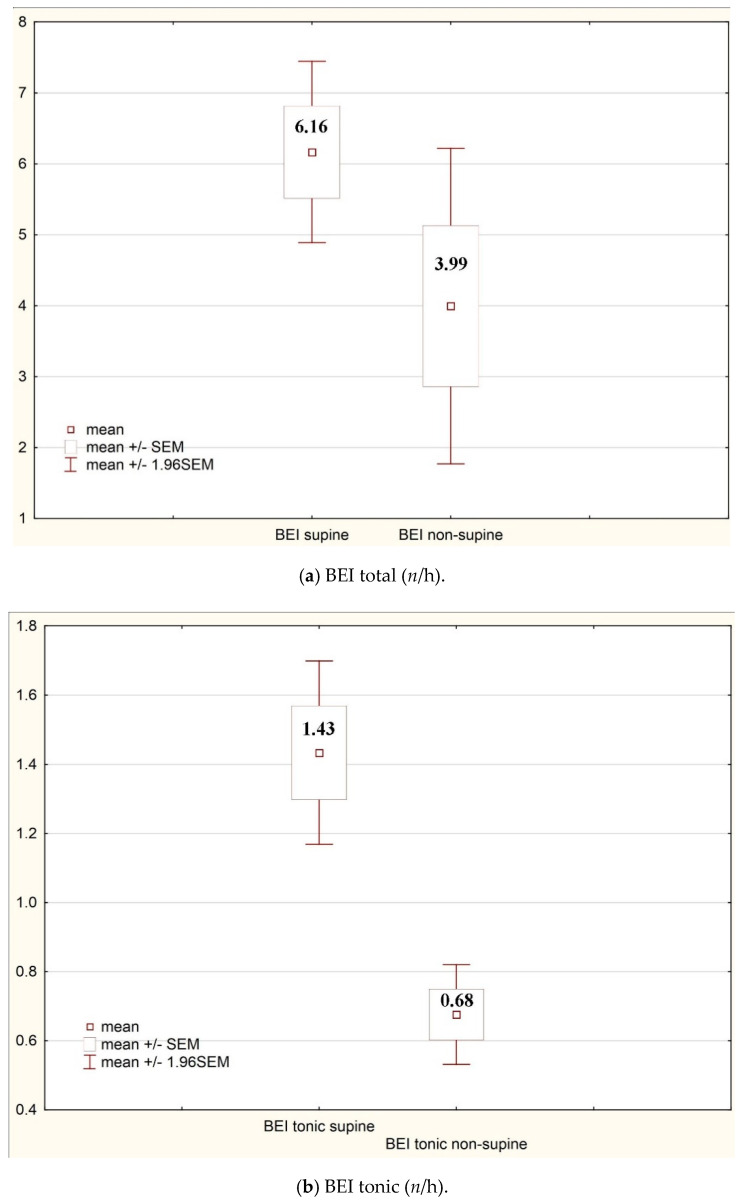
Impact of the body position on bruxism indices.

**Figure 2:**
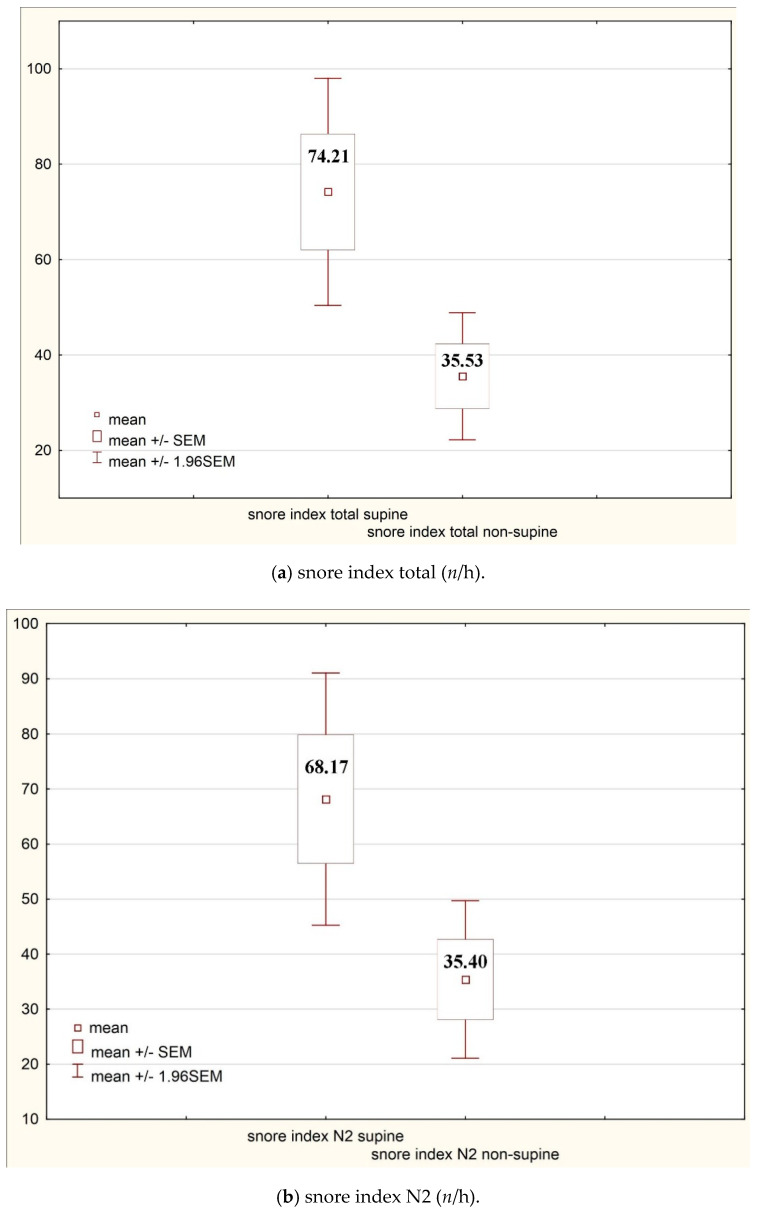

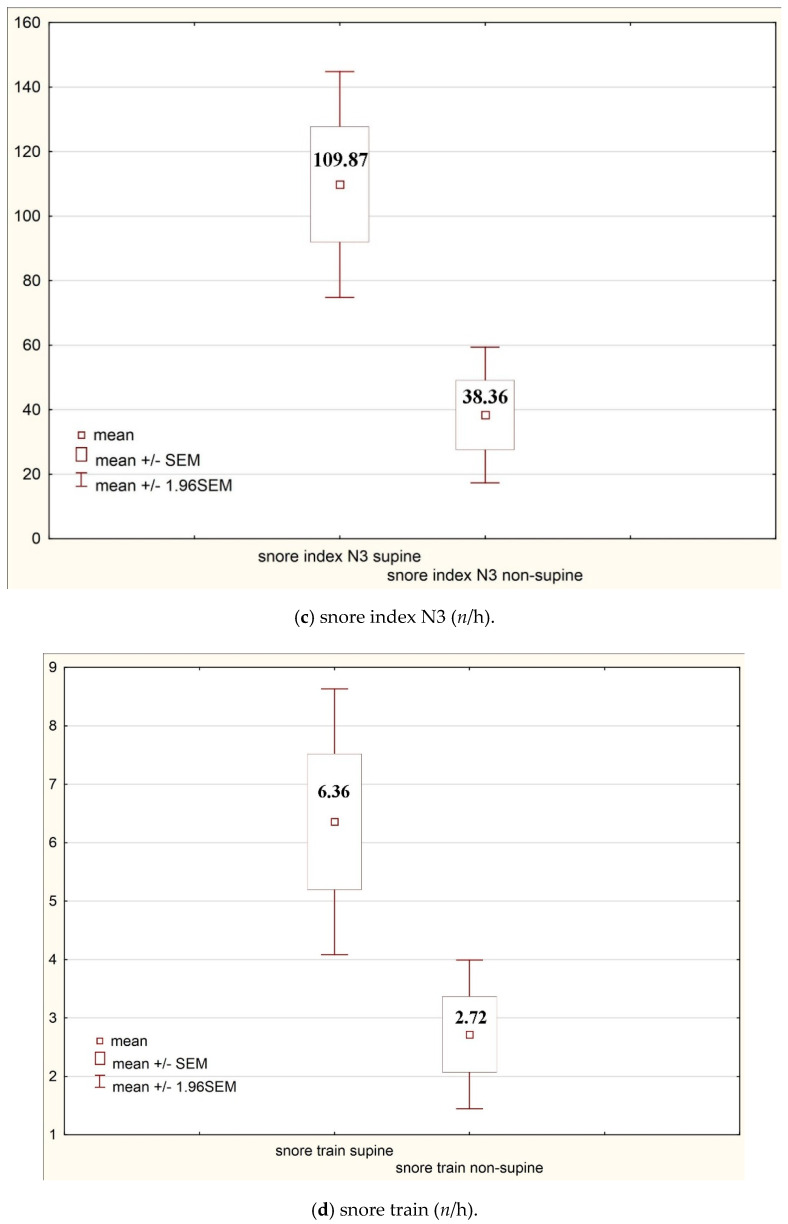

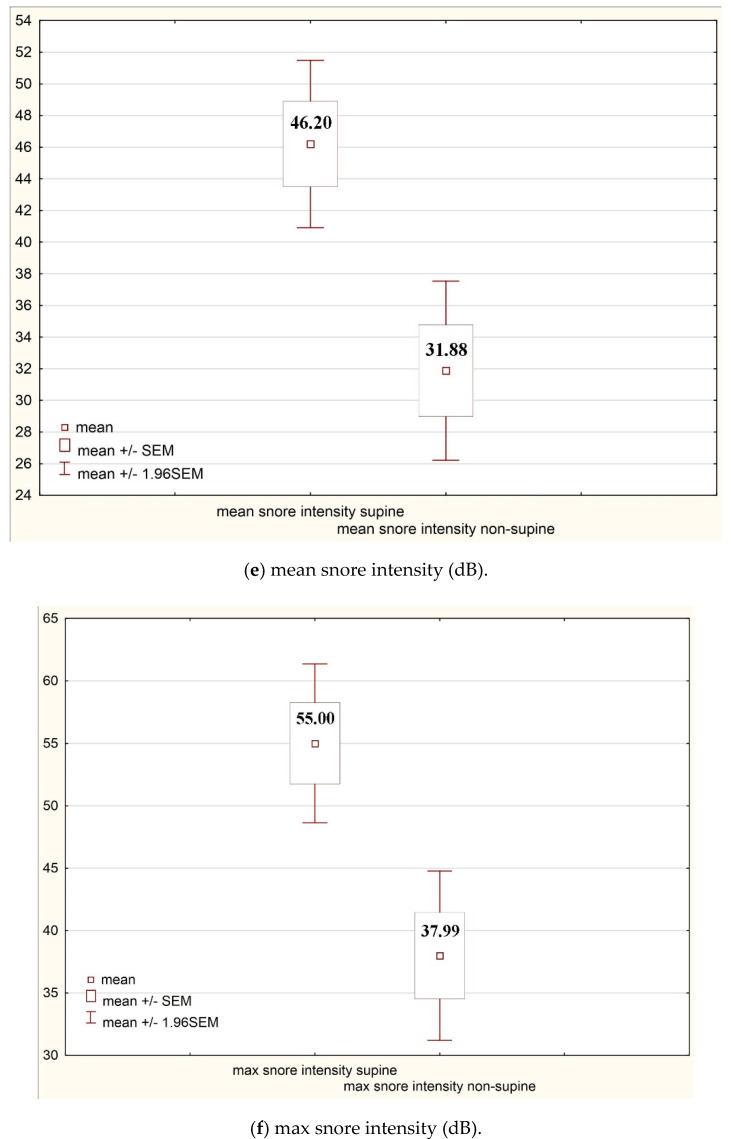

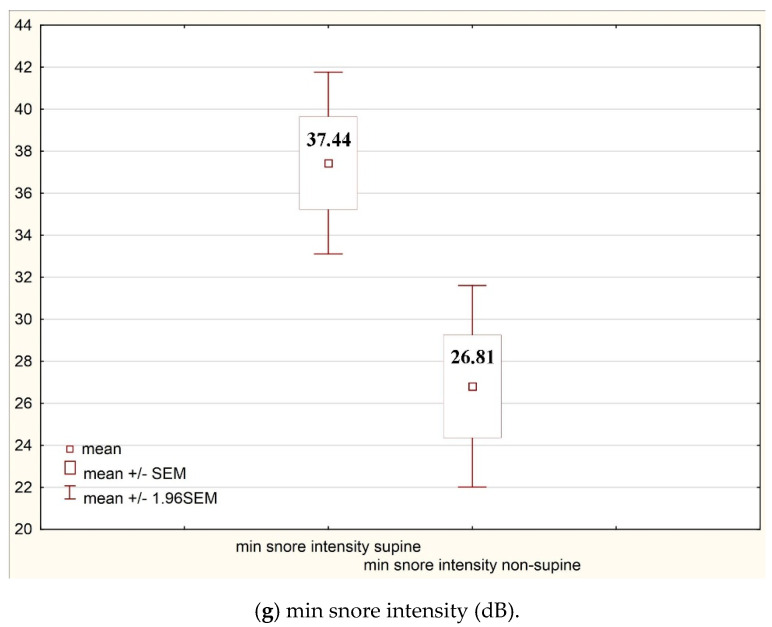
Impact of the body position on snore indices.

The statistically significant differences from Table 6 and Table 7 have been presented in Figure 1 and Figure 2.

In order to verify the independence of the obtained relationships between snore intensity parameters and BEI, regression analysis was performed, obtaining the following models:

Maximum snore intensity = 1.83 BEI + 0.51 N3 + 8.20 mean desaturation drop +0.19 maximum heart rate;

Mean snore intensity supine = 1.51 BEI + 5.09 AHI + 1.77 sleep stage N3 + 1.33 sleep stage REM;

Maximum snore intensity supine = 1.82 BEI + 5.81 AHI + 4.69 sleep stage N3 − 4.27 mean SpO_2_ + 0.37 maximum heart rate;

Minimum snore intensity supine = 1.45 sleep stage N3 + 1.16 sleep stage REM − 1.10 mean SpO_2_.

Regression analysis showed that the relationship between snore intensity parameters and BEI is independent for maximum snore intensity, mean snore intensity supine, and maximum snore intensity supine. Higher BEI, higher percentage of N3 sleep stage, higher mean desaturation drop, and higher maximum heart rate are independently associated with higher maximum snore intensity. Higher mean snore intensity supine is independently influenced by higher BEI, higher AHI, higher percentage of N3 sleep stage, and higher percentage of REM sleep stage. Higher maximum snore intensity supine is the result of higher BEI, higher AHI, higher percentage of N3 sleep stage, lower mean SpO_2_, and higher maximum heart rate, independently of each other.

Regression analysis also showed that BEI is not an independent predictor of minimum snore intensity supine. Independent factors associated with higher minimum snore intensity supine are higher percentage of N3 sleep stage, higher percentage of REM sleep stage and lower mean SpO_2_.

## 4. Discussion

The findings of the present study demonstrate the common coexistence of sleep bruxism and simple snoring. Sleep bruxism is classified by the ICSD-3 as a sleep-related movement disorder [41] and confirmed by several studies as decreasing the quality of life [43,44] and increasing the risk of negative consequences for oral health [15,45] and general health condition [18,19,20,23,24]. The consensus authors, Lobbezoo et al., suggested that bruxism is only a risk factor that increases the probability of a harmful effect but does not guarantee it [15,46]. Sometimes, bruxism may have a protective effect; for example, in a study by Ohmure et al., the episodes of rhythmic masticatory muscle activity (RMMA) were significantly higher in the 20-min period after acidic infusion causing salivation that protects against chemical tooth wear which occurs in patients with gastroesophageal reflux [47]. Similarly, the role of snoring, especially among people without sleep apnea, remains unclear. Some authors have demonstrated that snoring causes a number of severe physical implications such as excessive daytime sleepiness [4], carotid artery atherosclerosis [5], stroke [6], cardiovascular diseases [7], metabolic syndrome [8], and increased all-cause mortality [9]. Most of these are exactly in line with the consequences of sleep bruxism. Although studies on simple snoring and sleep bruxism have been conducted by many authors, their implications are still insufficiently explored and it remains unclear as to what extent these conditions can be attributed to severe health conditions. However, it is worth noting that SB and SS commonly coexist.

The results of our study confirm the statistically significant correlation between SB and SS. Snore intensity (loudness) in supine sleep position was found to be positively correlated with BEI total and phasic. Such a correlation was also found in the non-supine position for phasic bruxism and snoring, but as far as we know, no previous research has investigated this relationship. Palinkas et al. suggested that individuals with SB experience an increased amount of habitual snoring during sleep. The authors recorded the number of inspiratory, expiratory, and mixed snores but did not evaluate the loudness of snores [48]. In the current study, we examined the relationship between SS and SB and their parameters (intensity, frequency, types, and sleep position). Snore intensity was positively correlated with phasic bruxism subtype. Phasic bruxism was previously linked with hypoxic events due to its probable protective features [19]. Thus, the presence of positive correlation between phasic bruxism and snore intensity in current non-apneic study group suggest that simple snoring may have adverse effect on oxygen metabolism and intensive snoring could serve as a marker of mild obstruction. Therefore, the relationship between the simple snoring and sleep bruxism is investigated in the current work. Most earlier studies of our research team focused on the relationship between sleep bruxism and obstructive sleep apnea. For example, we confirmed the association between SB and mild and moderate stages of OSA [19]. Recently, our team showed that the single-nucleotide polymorphism (SNP) genetically affects the relationship between SB and OSA (HTR2A serotonin receptor encoding gene, rs2770304 SNP) [17]. SB and SS are considered as risk factors for negative health consequences [3,15] therefore the coexistence of them warrants further research by clinicians.

In this study, we showed that sleep position affects both snoring and bruxism indices. Statistically significant differences were found when comparing snore index, snore intensity, and the frequency of snore trains in supine and non-supine positions, and snoring was observed to be more frequent in the supine position. This result complies well with a previous study by Nakano et al. which demonstrated that most of the non-apneic snorers snored less in the lateral sleep position [49]. Taking under consideration, risk factors for snoring and obstructive sleep apnea are similar and include nasal congestion, obstruction of the upper airways, increased BMI, intake of alcohol, drug, or tobacco, and male gender [3]. Moreover, lateral sleep position was associated with OSA severity by many authors [50]. That gives the hypothesis, that supine sleep position in non-apneic patients which is associated with increased snore intensity could lead at least to development of OSA as a part of the continuum of disease predilection [3].

This paper also reports the impact of sleep position on SB, which has not been explained in the literature. SB was statistically more frequent in the supine position, but this was true only in the case of total and tonic bruxism. The difference between the prevalence of phasic bruxism in supine and non-supine positions was irrelevant. On the other hand, phasic bruxism was more frequent in the non-supine position, associated with desaturation. These findings go beyond previous reports that described the relationship between the sleep position and the severity of bruxism. In one of them, bruxism severity was estimated from the frequency of RMMA episodes [51], and the results showed that patients who were suffering from SB had 74% of RMMA and swallowing events in the supine position compared with 23% in the lateral decubitus position. An apparent limitation of the aforementioned research is a small study group (nine patients with SB and seven normal individuals in the control group). In another study by Phillips et al., bruxism activity was found to be higher in individuals with increased AHI, which indicated a correlation between clenching index and AHI. In addition, it was observed that clenching index and AHI were higher in the supine position than in the lateral decubitus position [27]. This study also involved a small population (consisting of 24 individuals). In both studies, the patients underwent polysomnography. The findings of the present study also support the hypothesis that the phasic and tonic types of bruxism exhibit different features and roles. Phasic bruxism was linked with sleep-related breathing disorders by some authors. Tan et al. indicated the predominant role of phasic bruxism in OSA patients and suggested that RMMA possibly had a protective effect against respiratory-related arousals [52]. Similar findings were shown in the study by Hosoya et al., in which phasic SB correlated positively with OSA, microarousals, and oxygen desaturation [28]. Our analysis proved that phasic bruxism contributed to oxygen desaturation events, independent of sleep position. Generally, SB events were more frequently observed in the supine position and, hence, the positive correlation of phasic SB with ODI confirms its predominant role in desaturation events. The role of tonic SB in SRBDs was emphasized in a study by Smardz et al., which showed a significant relationship between the tonic EMG pathways in SB episodes and SRBDs [53]. However, this issue should be further explored.

An interesting result of our study is the positive correlation between ODI and BEI in the non-supine position (total, mixed, and phasic), while the correlation of ODI with tonic bruxism was irrelevant. The positive correlation between phasic bruxism and ODI was demonstrated in our prior research [19], in which mild and moderate obstructive sleep apnea was linked with sleep bruxism. To investigate the relationship between sleep bruxism and non-apneic hypoxia, we designed the current research. Our study group involved only individuals without obstructive sleep apnea, and thus the increased BEI observed in the subjects can be considered as a physiological response to low blood oxygen level (expressed as increased ODI). It is worth noting that this positive correlation was only observed for the non-supine sleep position, whereas total and tonic bruxism was predominant in the supine position with respect to BEI and sleep position association in total. This result suggests the protective effect of SB against hypoxia independent of sleep position. The association between these two conditions in the absence of sleep-disordered breathing was also investigated by Dumais et al. Their results suggested that transient hypoxia was probably a factor linked with the onset of RMMA, regardless of concomitant sleep arousal or body movements [54]. However, there were some limitations in the study such as insufficient sample size (22 participants) and the use of ambulatory, home PSG.

Regarding sleep latency, a positive correlation was found between BEI non-supine and the two phenotypes of sleep bruxism: mixed and phasic non-supine. The presence of a long SL, frequent awakenings, or prolonged WASO is considered as evidence of insomnia [55], so our findings signal the need for additional studies to understand more about this relationship. Bruxism episodes are more frequent during light sleep and are associated with arousals [56,57,58]; therefore, prolonged SL, as an element of sleep architecture with wake/N1 sleep stage transition [59], may provoke bruxism events. Our results are consistent with other studies indicating insomnia as a risk factor for bruxism. For instance, a study conducted by Ahlberg et al. investigated self-reported bruxism and perceived sleep difficulties [60]. An apparent limitation of this survey-based research was the lack of objective assessment of the reported complaints. Furthermore, a statistically significant relationship was demonstrated by Maluly et al. This population-sampled study showed a positive association between SB and insomnia, higher degree of schooling, and a normal/overweight BMI [58]. On the other hand, in the study by Kishi et al., sleep latency was shorter in bruxers compared with controls [61]. These suggest that further research with the instrumental approach is needed to confirm our novel findings.

Our research indicated a negative correlation between BEI total, phasic, and mixed subtypes and minimum heart rate. Moreover, based on sleep position, positive correlations were observed between mean and minimum heart rate, and BEI total, mixed, and phasic in the non-supine position. Individuals suffering from SB had significantly decreased mean and minimum heart rate than others, independent of bruxism severity. These results are identical to that of a previous study by Haraki et al., which also demonstrated that mean heart rate during sleep was lower in individuals with high-RMMA (BEI > 5.7 *n*/h) frequency, but the heart rate variability did not differ between the SB group and controls [62]. The authors implicated that these findings may suggest that a more recuperative function occurs to maintain normal sleep in young individuals or that the arousals related to SB events are neither frequent nor intense enough to cause any change in the sleep macrostructure. In an original study by Huynh et al., the association between the incidence of RMMA and autonomic cardiac activity across sleep cycles was demonstrated; the authors showed that in moderate-to-severe sleep bruxism, increase in sympathetic activity precedes the onset of SB [63]. A review paper by Lavigne et al. also indicated this relationship [64]. Furthermore, it has been previously reported in the literature that SB is linked with increased sympathetic tone and heart rate variability [65].

The results of our study suggest that the sympathovagal balance in SB subjects is expressed by a significantly decreased heart rate, probably as the response to the increase in heart rate preceding microarousal or an SB episode. Having low heart rate in patients with SB is the implication of this self- regulating mechanism and could lead to insufficient blood flow, fatigue, and other bradycardia consequences.

Regression analysis has showed that BEI, increase of N3 sleep, and hypoxia expressed as higher mean desaturation drop are independently associated with snore intensity, whereas supine snore intensity is influenced by BEI, AHI, increase of N3 sleep, higher heart rate, and lower oxygen saturation, all independent of one another. Summarizing, sleep bruxism, hypoxia, and sleep architecture may influence snore intensity in non-apneic adults.

One of the strengths of our study was that full-night polysomnography was performed to diagnose SS, SB, and respiratory events in the participants. To our knowledge, this study is the only study to investigate the association between BEI, sleep position, and snoring in patients without OSA. These findings include probable key component in future attempts to lower snoring by treating SB or influencing risk for OSA by modifying SB and SS intensity. Moreover, a large, heterogenous group was analyzed in the study, which increased the research relevance. However, the study had some limitations. First, lack of parity (38 men and 91 women) and a narrow age range of participants restricted study interpretation. The features displayed by younger SB patients differed from those of the elderly. Apparently, sample bias of the study group involved lack of probability sampling. We have decided to include in our study only patients with reported snoring and/or probable bruxism. Moreover, hypertension, diabetes, increased BMI, neck circumference, and the Mallampati Score that are known as risk factors for simple snoring and/or sleep bruxism were not evaluated and included in the exclusion criteria. Second, prior research studies on the topic are limited and the theoretical foundation is poor. However, we considered this limitation as an opportunity to identify new gaps in the prior literature and highlight the need for further development in this area of study. Third, for bruxism assessment, electrode leads were inserted into EMG inputs of the Nox A1 device. The same inputs are used for leg EMG, thus device construction limited ability to conduct an analysis of periodic limb movement.

## 5. Conclusions

Overall, our results showed the association between sleep bruxism and simple snoring. Snore intensity was positively correlated with phasic bruxism, both in supine and non-supine sleep positions, which is an important and novel finding. In addition, body position affects the intensity of both snoring and bruxism. Results of current study provided evidence for the relationship between hypoxia and sleep bruxism in individuals with simple snoring. Moreover, data also indicated a negative correlation between phasic bruxism and minimum heart rate, as the effect of sympathovagal homeostasis. Looking forward, further research is needed to assess clinical implications of both sleep bruxism and simple snoring for patients without obstructive sleep apnea.

## Figures and Tables

**Table 1:** Bruxism and respiratory parameters—polysomnographic characteristics of the study group.

Parameter	Mean	SD
BEI (*n*/h)	4.39	3.33
Phasic (*n*/h)	2.49	2.74
Tonic (*n*/h)	1.20	1.01
Mixed (*n*/h)	0.75	0.66
AHI (*n*/h)	2.00	1.33
ODI (*n*/h)	2.38	1.74
Snore (% of TST)	5.01	9.78
Snore index (*n*/h)	61.64	105.82
TST (min)	429.68	56.45
SL (min)	22.83	21.60
WASO (min)	35.17	36.57
SE (%)	86.71	10.36
N1 (% of TST)	3.52	4.02
N2 (% of TST)	48.3	8.46
N3 (% of TST)	24.22	8.00
REM (% of TST)	23.97	6.06
Arousals (*n*/h)	3.28	2.39
Mean SpO_2_ (%)	94.86	1.58
Min SpO_2_ (%)	89.56	5.29
SpO_2_ < 90% (%)	0.70	3.20
Mean desaturation drop (%)	3.13	0.83
Mean heart rate (*n*/min)	61.26	9.84
Max heart rate (*n*/min)	99.29	19.11
Min heart rate (*n*/min)	48.69	7.21

BEI, bruxism episode index; AHI, apnea–hypopnea index; ODI, oxygen desaturation index; TST, total sleep time (min); SL, sleep latency; WASO, wake after sleep onset; SE, sleep efficiency; N1, sleep stage 1; N2, sleep stage 2; N3, sleep stage 3; REM, rapid eye movement sleep stage; mean SpO_2_, mean oxygen saturation (%); SpO_2_ < 90%, time with oxygen saturation < 90% (% of TST).

**Table 2:** Mean bruxism and snore parameters differentiated on the basis of body position.

Parameter	Mean	SD	Parameter	Mean	SD
BEI supine (*n*/h)	6.16	7.40	Snore index supine (*n*/h)	74.21	138.05
BEI mixed supine (*n*/h)	0.85	0.89	Snore index non-supine (*n*/h)	35.53	77.18
BEI phasic supine (*n*/h)	3.52	4.92	Snore index N1 (*n*/h)	67.97	84.82
BEI tonic supine (*n*/h)	1.43	1.54	Snore index N1 supine (*n*/h)	67.32	102.80
BEI non-supine (*n*/h)	3.99	12.88	Snore index N1 non-supine (*n*/h)	58.55	119.32
BEI mixed non-supine (*n*/h)	0.72	2.19	Snore index N2 (*n*/h)	60.28	108.25
BEI phasic non-supine (*n*/h)	2.61	10.77	Snore index N2 supine (*n*/h)	68.17	132.57
BEI tonic non-supine (*n*/h)	0.68	0.84	Snore index N2 non-supine (*n*/h)	35.40	82.84
Mean snore intensity (dB)	54.13	25.45	Snore index N3 (*n*/h)	90.06	168.28
Max snore intensity (dB)	65.38	31.23	Snore index N3 supine (*n*/h)	109.87	202.72
Min snore intensity (dB)	43.78	20.97	Snore index N3 non-supine (*n*/h)	38.36	122.04
Mean snore intensity supine (dB)	46.20	30.62	Snore index REM (*n*/h)	42.86	108.45
Max snore intensity supine(dB)	55.00	36.81	Snore index REM supine (*n*/h)	44.35	117.10
Min snore intensity supine (dB)	37.44	25.05	Snore index REM non-supine (*n*/h)	24.61	72.54
Mean snore intensity non-supine (dB)	31.88	32.81	Snore train supine (*n*/h)	6.36	13.18
Max snore intensity non-supine (dB)	37.99	39.28	Snore train non-supine (*n*/h)	2.72	7.36
Min snore intensity non-supine (dB)	26.81	27.76			

BEI, bruxism episode index; N1, sleep stage 1; N2, sleep stage 2; N3, sleep stage 3; REM, rapid eye movement sleep stage.

**Table 3:** Polysomnographic indices correlated with bruxism and the body position.

Body Position	Bruxism Indices	AHI (*n*/h)	ODI (*n*/h)	Snore (% of TST)	SL (min)	Arousals (*n*/h)	Mean Heart Rate (*n*/min)	Max Heart Rate (*n*/min)	Min Heart Rate (*n*/min)
Total	Average BEI (*n*/h)	0.08	0.09	−0.02	−0.00	**0.23**	−0.16	0.04	**−0.25**
	Phasic (*n*/h)	0.10	0.10	−0.01	0.00	0.10	−0.15	0.09	**−0.25**
	Tonic (*n*/h)	0.08	0.07	0.03	−0.05	**0.27**	−0.06	−0.11	−0.03
	Mixed (*n*/h)	−0.09	−0.06	−0.09	−0.01	**0.35**	−0.09	0.05	**−0.22**
Supine	Average BEI (*n*/h)	0.07	0.03	−0.07	−0.02	0.15	−0.08	−0.02	−0.12
	Phasic (*n*/h)	0.12	0.10	−0.06	0.01	0.04	−0.10	0.01	−0.17
	Tonic (*n*/h)	0.08	0.10	0.02	−0.03	**0.25**	−0.03	−0.11	−0.04
	Mixed (*n*/h)	0.12	0.10	−0.03	0.00	**0.36**	−0.14	−0.04	**−0.26**
Non- supine	Average BEI (*n*/h)	0.12	**0.29**	−0.05	**0.31**	−0.01	**0.20**	0.09	**0.21**
	Phasic (*n*/h)	0.13	**0.29**	−0.04	**0.30**	−0.02	**0.20**	0.09	**0.21**
	Tonic (*n*/h)	0.05	0.04	−0.09	0.03	0.09	−0.01	−0.05	0.02
	Mixed (*n*/h)	0.07	**0.25**	−0.08	0.32	−0.01	**0.22**	0.11	**0.23**

BEI, bruxism episode index; AHI, apnea–hypopnea index; ODI, oxygen desaturation index; TST, total sleep time (min); SL, sleep latency. The values in the table represent correlation coefficients. Statistically significant correlations are marked in bold (*p* < 0.05).

**Table 4:** Results for heart rate in bruxers and non-bruxers, differentiated on the basis of bruxism severity.

Parameter	BEI < 2 (*n*/h)		BEI > 2 (*n*/h)			BEI < 4 (*n*/h)		BEI > 4 (*n*/h)		
	Mean	SD	Mean	SD	*p*	Mean	SD	Mean	SD	*p*
Mean heart rate (*n*/min)	64.47	11.25	60.25	9.18	0.04	62.95	9.88	59.26	9.49	0.03
Max heart rate (*n*/min)	101.90	18.41	98.47	19.34	0.39	99.46	19.25	99.10	19.09	0.92
Min heart rate (*n*/min)	51.00	8.36	47.96	6.69	0.04	49.87	8.05	47.29	5.84	0.04

BEI, bruxism episode index.

**Table 5:** Correlation between snore intensity parameters and bruxism episode index.

Parameter	BEI (*n*/h)	Phasic (*n*/h)	Tonic (*n*/h)	Mixed (*n*/h)
Snore index (*n*/h)	0.03	0.06	0.03	−0.12
Mean snore intensity (dB)	0.17	0.17	0.02	0.15
Max snore intensity (dB)	**0.21**	**0.22**	0.02	0.15
Min snore intensity (dB)	0.15	0.13	0.04	**0.18**
Mean snore intensity supine (dB)	**0.19**	**0.22**	0.01	0.09
Max snore intensity supine(dB)	**0.21**	**0.24**	0.03	0.09
Min snore intensity supine (dB)	**0.18**	**0.21**	0.02	0.10
Mean snore intensity non-supine (dB)	0.15	**0.21**	−0.08	0.03
Max snore intensity non-supine (dB)	0.17	**0.23**	−0.09	0.03
Min snore intensity non-supine (dB)	0.14	**0.19**	−0.09	0.04

BEI, bruxism episode index. The values in the table represent correlation coefficients. Statistically significant correlations are marked in bold (*p* < 0.05).

**Table 6:** Impact of the body position on bruxism indices.

	Supine		Non-Supine		
BEI (*n*/h)	Mean	SD	Mean	SD	*p*
Total	**6.16**	**7.04**	**3.99**	**12.88**	**0.04**
Mixed	0.85	0.89	0.72	2.19	0.53
Phasic	3.52	4.92	2.61	10.77	0.37
Tonic	**1.43**	**1.54**	**0.68**	**0.84**	**0.00**

BEI, bruxism episode index. Statistically significant differences are marked in bold (*p* < 0.05).

**Table 7:** Impact of the body position on snore indices.

	Supine		Non-Supine		
Parameter	Mean	SD	Mean	SD	*p*
Snore index total (*n*/h)	**74.21**	**138.05**	**35.53**	**77.18**	**0.00**
Snore index N1 (*n*/h)	67.32	102.80	58.55	119.32	0.52
Snore index N2 (*n*/h)	**68.17**	**132.57**	**35.4**	**82.84**	**0.01**
Snore index N3 (*n*/h)	**109.87**	**202.72**	**38.36**	**122.04**	**0.00**
Snore index REM (*n*/h)	44.35	117.10	24.61	72.54	0.10
Snore train (*n*/h)	**6.36**	**13.18**	**2.72**	**7.36**	**0.00**
Mean snore intensity (dB)	**46.20**	**30.62**	**31.88**	**32.81**	**0.00**
Max snore intensity (dB)	**55.00**	**36.81**	**37.99**	**39.28**	**0.00**
Min snore intensity (dB)	**37.44**	**25.05**	**26.81**	**27.76**	**0.00**

N1, sleep stage 1; N2, sleep stage 2; N3, sleep stage 3; REM, rapid eye movement sleep stage. Statistically significant differences are marked in bold (*p* < 0.05).
